# Localization of SH3PXD2B in human eyes and detection of rare variants in patients with anterior segment diseases and glaucoma

**Published:** 2012-03-26

**Authors:** Mao Mao, Frances Solivan-Timpe, Ben R. Roos, Robert F. Mullins, Thomas A. Oetting, Young H. Kwon, Peter M. Brzeskiewicz, Edwin M. Stone, Wallace L.M. Alward, Michael G. Anderson, John H. Fingert

**Affiliations:** 1 Department of Molecular Physiology and Biophysics, The University of Iowa, Iowa City, IA; 2 Department of Ophthalmology and Visual Sciences, The University of Iowa, Iowa City, IA; 3 Howard Hughes Medical Institute, Iowa City, IA

## Abstract

**Purpose:**

Analysis of mutant mouse strains and linkage analysis with human families have both demonstrated that mutations influencing the podosomal adaptor protein SH3 and PX domains 2B (SH3PXD2B) can result in a congenital form of glaucoma. Here, we use immunohistochemistry to describe localization of the SH3PXD2B protein throughout the adult human eye and test whether sequence variants in *SH3PXD2B* occur in multiple other forms of glaucoma.

**Methods:**

In immunohistochemical experiments, cryosections of human donor eyes were evaluated for SH3PXD2B immunoreactivity with a polyclonal antibody. In genetic experiments, exon sequences of *SH3PXD2B* from patients with primary congenital glaucoma (n=21), Axenfeld-Rieger syndrome (n=30), and primary open angle glaucoma (n=127) were compared to control subjects (n=89). The frequency of non-synonymous *SH3PXD2B* coding sequence variants were compared between patient cohorts and controls using Fisher’s exact test.

**Results:**

Varying intensities of SH3PXD2B immunoreactivity were detected in almost all ocular tissues. Among tissues important to glaucoma, immunoreactivity was detected in the drainage structures of the iridocorneal angle, ciliary body, and retinal ganglion cells. Intense immunoreactivity was present in photoreceptor inner segments. From DNA analysis, a total of 11 non-synonymous variants were detected. By Fisher’s Exact test, there was not a significant skew in the overall frequency of these changes in any patient cohort versus controls (p-value >0.05). Each cohort contained unique variants not detected in other cohorts or patients.

**Conclusions:**

SH3PXD2B is widely distributed in the adult human eye, including several tissues important to glaucoma pathogenesis. Analysis of DNA variants in three forms of glaucoma detected multiple variants unique to each patient cohort. While statistical analysis failed to support a pathogenic role for these variants, some of them may be rare disease-causing variants whose biologic significance warrants investigation in follow up replication studies and functional assays.

## Introduction

The glaucomas are a leading cause of blindness worldwide [1]. All forms of glaucoma ultimately share a clinically recognizable form of progressive optic nerve degeneration, with several additional pathologic features often present in distinct forms of the disease [2]. There is a significant genetic contribution to the pathogenesis of most forms of glaucoma and while several loci associated with glaucoma have been mapped [3], known mutations only account for a small fraction of disease. Mutations in myocilin and optineurin are responsible for approximately 5% of primary open angle glaucoma (POAG) [4]. WD repeat domain 36 (*WDR36*) [5], neurotrophin 4 (*NTF4*) [6], ankyrin repeat and SOCS box-containing 10 (*ASB10*) [7], and TANK-binding kinase 1 (*TBK1*) [8] are other genes that have also been reported to be glaucoma-causing genes, but are controversial or have not yet been widely replicated. Similarly, genes have been discovered that cause primary congenital glaucoma (PCG), cytochrome P450, family 1, subfamily B, polypeptide 1 (*CYP1B1*) [9] and latent transforming growth factor beta binding protein 1 (*LTBP2*) [10], and Axenfeld-Rieger syndrome, paired-like homeodomain 2 (*PITX2*) [11] and forkhead box C1 (*FOXC1*) [12,13]. Mutations in *CYP1B1* are responsible for 10%–15% of simplex PCG cases [14-16], while mutations in *LTBP2* have only been reported in PCG families from Pakistan. It has been estimated that mutations in *PITX2* and *FOXC1* are associated with 25%–30% of cases of Axenfeld-Rieger syndrome in the United States [17], although these numbers vary significantly between patient populations. Nonetheless, these data indicate that many more disease-causing genes for these conditions have not yet been identified. Recent genome-wide association studies of primary open angle glaucoma have begun to identify genetic factors that each contribute small risk for disease, including caveolin 1 and 2 (*CAV1/CAV2*) [18] cyclin-dependent kinase inhibitor 2B antisense RNA 1 (*CDKN2B-AS1*) [19], and transmembrane and coiled-coil domains 1 (*TMCO1*) [19]. Risk alleles from these genes (and others) may combine to lead to the development of some cases of glaucoma. Many more of these risk alleles are likely to be discovered by larger glaucoma genome-wide association study (GWAS) that are currently underway.

Another approach to discover glaucoma genes is by studying the eyes of inbred mice. Recently, these investigations identified the SH3 and PX domains 2B (*SH3PXD2B*) gene as a potential glaucoma-causing gene [20-22]. The *nee* strain of mice is a spontaneously arising mutant that exhibits several glaucomatous defects, including developmental malformations of the iridocorneal angle, elevated intraocular pressure, and optic nerve degeneration [22]. We have recently identified the genetic basis of the *nee* phenotype as a 1-bp *Sh3pxd2b* deletion that is predicted to result in a frame-shift and premature stop codon [21]. Independently, Iqbal et al. [20] used linkage analysis and characterization of a mouse mutation generated via gene-trap to link *SH3PXD2B* with Frank-Ter Haar syndrome, a condition often involving congenital glaucoma [23,24]. Thus, two independent lines of investigation have suggested that severe loss-of-function mutations in *SH3PXD2B* could contribute to developmental forms of glaucoma. It remains unknown what role, if any, that hypomorphic alleles of *SH3PXD2B* might have.

We have tested the role of *SH3PXD2B* in glaucoma pathogenesis. The *Sh3pxd2b* mutant mice have a homozygous 1 base pair deletion in the *Sh3pxd2b* gene and develop congenital glaucoma with features similar to Axenfeld-Rieger syndrome. The mice have congenital craniofacial abnormalities and peripheral anterior synechiae that mimic the maxillary hypoplasia and iridocorneal angle abnormalities that characterize Axenfeld-Rieger syndrome [21,22]. As a result, we have tested the role of *SH3PXD2B* in Axenfeld-Rieger syndrome by testing a cohort of patients for disease-causing mutations. Given the role of *SH3PXD2B* in syndromic congentital glaucoma associated with Frank-Ter Haar syndrome and the early onset glaucoma phenotype in the *Sh3pxd2b* mutant mice, we also tested a cohort of primary congenital glaucoma patients for disease-causing mutations in *SH3PXD2B*. We similarly tested a cohort of adult-onset primary open angle glaucoma (POAG) patients to determine if variants in *SH3PXD2B* have a role in the pathogenesis of this more common form of glaucoma. We also report localization of SH3PXD2B protein throughout the normal human eye using immunohistochemistry. The results demonstrate that SH3PXD2B is broadly expressed in many ocular tissues important to glaucoma and that the *SH3PXD2B* gene harbors rare variants that may be important in the pathophysiology of glaucoma.

## Methods

### Immunohistochemistry

Human donor eyes were obtained from the Iowa Lions Eye Bank (Iowa City, IA) following informed consent from the donors’ families. The average death-to-preservation time for the eyes used in this study was 5.75 h (range 3.75 to 8.1 h). Immunohistochemistry was performed on tissue from two normal eyes of donors with ages ranging from 61 to 88 years. Eyes were processed immediately on receipt. Lenses were removed, and tissues from the anterior and posterior poles were punched using disposable trephines, and punches were fixed with 4% paraformaldehyde in phosphate buffered saline (PBS) for 2 h. The removed lenses were fixed separately with a similar approach. After fixation, tissues were rinsed with PBS. Tissues were cryopreserved with sucrose gradient and embedded in Optimal Cutting Temperature embedding medium (Tissue-Tek O.C.T. Compound; Sakura Finetek, Torrance, CA) [25]. Anterior and posterior punches were cut at 6–8 µm thickness. The lenses were cut at 18–20 µm thickness. Sections were air dried for 30 min at room temperature, rehydrated in PBS for 5 min, and blocked with 10% goat serum, 3% BSA (BSA) in PBS for 1 h at room temperature. Sections were then incubated overnight at 4 °C with a rabbit anti-human SH3PXD2B polyclonal antibody (Millipore, Temecula, CA) diluted at 1:50 in 1% goat serum, 1% BSA in PBS. Adjacent sections incubated without the primary antibody were used as negative controls. After washes with 0.1% Tween-20 in PBS (3×10 min), sections were incubated with Alexa488-conjugated goat anti-rabbit antibody (Invitrogen, Carlsbad, CA) diluted at 1:200 in 1% goat serum, 1% BSA in PBS for 1 h at room temperature. Following washes, sections were incubated with To-Pro-3 (1:1000 dilution in PBS; Invitrogen, Carlsbad, CA) for 15 min at room temperature to stain nuclei. Sections were then washed several times in PBS, mounted (ProLong Gold; Invitrogen, Carlsbad, CA) and imaged with a confocal microscope (Zeiss LCM 510; Carl Zeiss MicroImaging, Inc., Thornwood, NY). Two eyes were examined and immunolabeling was repeated twice for each eye.

### Human subjects

All subjects enrolled in the study gave informed consent and the research was conducted with approval of the University of Iowa’s Internal Review Board. Twenty-one patients with primary congenital glaucoma had typical features of disease including a diagnosis before 3 years of age, open angles on gonioscopy, elevated intraocular pressure, buphthalmos, and Haab striae. Thirty patients with Axenfeld-Rieger syndrome had characteristic features of the condition including posterior embrotoxon, iris processes, correctopia, polycoria, redundant periumbilical tissue, and dental abnormalities. One hundred and twenty seven patients with POAG had excavation of their optic nerve head with resultant glaucomatous visual field loss in at least one eye. Glaucomatous optic nerves had cup-to-disc ratios of greater than 0.7 with thinning of the neural rim, asymmetry of the optic nerve cup-to-disc ratio of >0.2, or photographic documentation of progressive loss of the neural rim. Patients were 40 years of age or older at diagnosis and had open iridocorneal angles on gonioscopy (angle greater than Shaffer grade II). Patients were also required to have an IOP of greater than 21 mmHg on at least one occasion. Eighty-nine control subjects were a minimum of 50 years old and were examined and judged to have normal optic nerve head appearance and IOP ≤21 mmHg by board-certified ophthalmologists. All study subjects were examined by clinicians at the University of Iowa Hospitals and Clinics and ascertained in Iowa.

### Genetic analysis

DNA samples were prepared from peripheral blood samples extracted from patients in the clinic by standard procedures. The coding region of *SH3PXD2B* (NM_001017995) was PCR amplified using overlapping primer pairs in standard PCR reactions (Table 1). This assay encompass 97% of the coding sequence of the longest isoform of *SH3PXD2B* . Amplified DNA was scanned for mutations with a combination of single strand conformation polymorphism (SSCP) analysis and bi-directional DNA sequencing with an Applied Biosystems (ABI) model 3730 automated sequencer as previously described [8]. Those mutations that result in amino acid substitutions were evaluated using the blosum62 matrix, which provides an integer score for these substitutions that ranges from −4 to +3. More positive blosum62 scores indicate conservative amino acid changes that are less likely to be pathogenic, while more negative scores indicate less conservative substitutions that are more likely to cause disease [26].

**Table 1:** The coding region of *SH3PXD2B* was PCR amplified using overlapping primer pairs.

**Exon**	**Forward primer**	**Reverse primer**
2	GTCCCAGAGATTGGGAGACC	GAATGTAAGTCCAATTAAACTCTTTCC
3	AAATGTCCTAGATGATGTTTAGTGC	CAAGGGCTCTGGGAACTGTA
4	GGCACCACTCAGACCTACCC	GCACAAATTTTTATTGTTGAGCAT
5	CAAACAATTATCTTGCCTCAGC	TGCTTTACTTGGGGGTGGC
6	AATACATGGCAAGTCTGACTCG	GTTTGCCGAAAACTGAACGA
7	TGACTCCTGCTCTTTCATGC	GAGTTTCCAAATGTTTCATGTCC
8	TTCACTGGTACAGTGGCTGAAT	GCAACCCAGTATAGGCGATG
9	AAGGGCATCACGGGGATT	GTGAGGCCAGAGTCCCTGT
10A	TGTGATTCCCAGTAGGAGCA	TGCTGAGCAGCTCCTTCT
10B	GTGCCCTTGACTTGGATGG	GATGTGAGACGCCTTGAGC
11	CCCAGCTCAGGAATCTCATC	TGTGTGAGGGGCTAGTGGAC
12	GACACAGGGTCGCAGGAGT	GGGGAGAAGTAGGAGGTGATG
13A	CCAAACCATTCCATCTGCTG	GGAGCTGGGTCACCTCGT
13B	AGGACTCTTTGTATGTGGCCGTG	AAGCCAGCAAGGACCAGCGGG
13C	AACGCGTCGAGACCCAAC	GGGGTCTGAGATCTCCTCGTA
13D	ATGTCCTGAGGAAGGCATC	TTTTGTCAGGTTTGGGCTCT
13E	GTGATTTTGCCGATGATGC	TCTGGACTTCAAGAAGGGATTC
13F	GCCCATCTCCAAATCCAAAA	CCCTCCCCATCCAACAAG
13G	GACCAAGTCGACATCTGCAA	GCACGCTCTTAGACACAGGAT
13H	GGGCAAACAGGATGGTCT	GAGAAAAGGTTTGGCTTTTGG
13I	ACAGTGTGAAGGCCACGAAA	CCTGGAAGCTGCTGGTGT

## Results

### *SH3PXD2B* expression in human eyes

**Figure 1:**
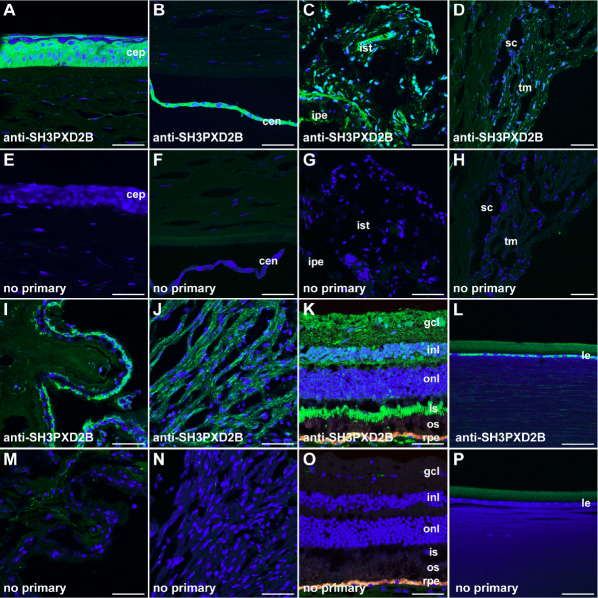
Localization of *SH3PXD2B* in human eyes. Immunohistochemistry labeling of SH3PXD2B on human eyes reveals localization of SH3PXD2B in most ocular cell types. (**A**-**D**, **I**-**L**) Cryosections were labeled with an anti-SH3PXD2B antibody (*Green*) and To-Pro-3, a nuclear counterstain (*blue*). (**E**-**H**, **M**-**P**) Negative controls omitting the primary antibody were performed on adjacent sections. (**A-B**, **E-F**) Cornea. (**C**, **G**) Iris. (**D**, **H**) Trabecular meshwork. (**I**, **M**) Ciliary processes. (**J**, **N**) Ciliary muscles. (**K**, **O**) Retina. (**L**, **P**) Lens. The orange-yellow color in **K** and **O** represents lipofuscin autofluorescence in the retinal pigment epithelium. cep, corneal epithelium; cen, corneal endothelium; ist, iris stroma; ipe, iris pigment epithelium; tm, trabecular meshwork; sc, Schlemm’s canal; gcl, ganglion cell layer; inl, inner nuclear layer; onl, outer nuclear layer; is, inner segment; os, outer segment. Scale bar=50 µm.

Based on several published microarray studies [27-33], in situ hybridization data from mice [20], and limited experiments with ocular tissues dissected from mice [22], *SH3PXD2B* is predicted to have a broad ocular expression. However, the immunolocalization of SH3PXD2B protein throughout the eye has not previously been examined. To characterize the distribution of SH3PXD2B protein in adult human eyes, immunofluorescent labeling was performed using a polyclonal antibody against human SH3PXD2B on cryosections of healthy human donors (Figure 1). Presence of SH3PXD2B immunoreactivity was demonstrated on multiple tissues in the eye, including the cornea, iris, trabecular meshwork, ciliary body, retina, and the lens. In the cornea, relatively strong immunostaining was observed in the cytoplasm of corneal epithelium (Figure 1A) and endothelium (Figure 1B), while there was definite but weak labeling of the keratocytes in the corneal stroma (Figure 1A,B). Similarly, wide distribution of SH3PXD2B was also found in the cytoplasm of all cell types of the iris and trabecular meshwork (Figure 1C,D). In the ciliary body (Figure 1I,J), strong labeling was detected in the non pigmented epithelium of the ciliary process and the ciliary muscle. Immunoreactivity of the pigmented epithelium of the ciliary process was less intense. In the retina (Figure 1K), the immunoreactivity was detected in most layers including the retinal ganglion layer, the main cell type affected during glaucoma. Interestingly, the strongest labeling of the retina was detected in the inner segment. Definite, but weak labeling of the lens epithelium and lens cortex were also observed (Figure 1L). No signal was detected in negative controls stained only with the secondary antibody (Figure 1E-H,M-P). These results demonstrate a broad distribution of SH3PXD2B in human eyes and support a possible role of SH3PXD2B in the pathogenesis of a variety of ocular diseases.

### DNA variations in *SH3PXD2B*

A total of 24 unique variations were detected in the *SH3PXD2B* gene including 11 non-synonymous coding sequence variations, 5 synonymous coding sequence variations, and 8 intron variations (Table 2).

**Table 2:** *SH3PXD2B* variants.

**Variations**	**BLOSUM62 matrix score**	**Located within protein domain**	**Primary congenital glaucoma n=21**	**Axenfeld-Reiger syndrome n=30**	**POAG n=127**	**Normal control subjects n=89**
**Non-synonymous coding sequence variations**						
Gly245Arg	−2	SH3 #2	0	0	1	0
Pro295Gln	−1	-	0	0	2	2
Arg356Gln	1	-	0	0	1	0
Glu396Lys	1	SH3 #2	1	0	0	0
Ala431Thr	0	-	0	1	0	0
Gly481Arg	−2	-	0	0	1	0
Pro571Leu	−3	-	0	1	0	0
Pro826Leu	−3	-	0	0	0	1
Ile832Val	3	-	0	0	1	0
Gly833Glu	−2	-	0	0	0	1
Glu834Lys	1	-	0	0	0	1
Total			1	2	6	5
**Synonymous coding sequence variations**						
Ala195Ala	-	-	0	0	1	0
Ser174Ser	-	-	0	0	3	1
Ser35Ser	-	-	16	22	90	61
Asp385Asp	-	-	1	0	0	1
Thr428Thr	-	-	1	0	0	1
**Intravening sequence variations**						
IVS3–28 a>g	-	-	3	1	9	4
IVS7–11 c>t	-	-	8	13	63	59
IVS7+50 t>c	-	-	0	0	3	1
IVS10–27 a>g	-	-	5	0	6	9
IVS11–9 t>c	-	-	1	1	0	0
IVS11–8 c>t	-	-	1	1	0	0
IVS11–7 g>t	-	-	0	0	2	1
IVS12–43 c>t	-	-	0	0	2	3

The SH3PXD2B protein has one segment with homology to a phosphoinositide binding Phox (PX) domain that extends from amino acid 7–125 and four src homology (SH3) domains that span amino acids 156–207, 225–277, 373–422, and 855–909 [34]. Non-synonymous mutations in *SH3PXD2B*, were not clustered within any particular functional domains of the gene. None of the detected non-synonymous codon variations (Table 2) were located in the PX domain, while one variation (Gly245Arg) was located in the second SH3 domain and another variation (Glu396Lys) was located within the third SH3 domain.

The detected SH3PXD2B variants were analyzed using the blosum62 matrix. Some amino acid substitutions are more deleterious to protein function than others and have more negative blosum62 scores. Each of the 11 non-synonymous coding sequence variants that we detected in SH3PXD2B was evaluated with the blosum62 matrix to estimate their potential effects on protein function (Table 2). Five of the 11 variants (Gly245Arg, Gly481Arg, Pro571Leu, Pro826Leu, Gly833Glu) had blosum62 scores of −2 or −3 which suggests that they may be harmful to protein function. It is notable that of these 11 variants, only one (Gly245Arg) is located within a known functional domain and has a negative blosom62 score.

When the frequencies of non-synonymous coding sequence variations were compared between the primary congenital glaucoma patients and control subjects, no significant difference was detected (p-value >0.99). Similar results were obtained for Axenfeld-Rieger syndrome (p-value >0.99) and POAG (p-value >0.76).

## Discussion

Animal models provide key resources for investigating the biologic pathways that lead from a gene defect to the development of disease. Studies of animal models have already facilitated the development of powerful diagnostic tests and effective therapeutic strategies, such as gene therapy for Leber Congenital Amaurosis caused by defects in the retinal pigment epithelium-specific protein 65kDa (*RPE65*) gene [35-37].

However, with respect to glaucoma, there are currently few mouse models that recapitulate the genotype and phenotype of human disease [38].

Multiple lines of evidence suggest that *SH3PXD2B* is relevant to human glaucoma. Loss of function mutations in *SH3PXD2B* have been linked to the form of congenital glaucoma occurring in Frank-Ter Haar syndrome [20] and *nee* mutant mice [21,39]. SH3PXD2B is an adaptor protein that has a vital role in the formation and function of podosome-like adhesions and interacts with other molecules that are important in maintenance of the extracellular matrix [21,39,40]. Podosomes have previously been observed in cells of the trabecular meshwork and are likely to regulate localization of matrix metalloproteinases capable of influencing outflow facility [41,42]. As such, it is plausible that SH3PXD2B may influence trabecular meshwork structure and function, facility of outflow, and intraocular pressure. Finally, we have shown with immunohistochemistry that SH3PXD2B is expressed in tissues of the human eye that are important in the glaucoma including the trabecular meshwork, ciliary body, and retina. Prior studies of SH3PXD2B showed that loss of function mutations are associated with a congenital form of glaucoma as part of Frank-Ter Haar syndrome, suggesting that we might find similar defects in a cohort of primary congenital glaucoma patients and possibly hypomorphic alleles in other forms of human glaucoma. Based on these observations, we set out to test cohorts of glaucoma patients for mutations in the *SH3PXD2B* to determine if the same defects that cause glaucoma in the *Sh3pxd2b* mutant mice are responsible for human disease.

We detected 14 instances of 11 non-synonymous *SH3PXD2B* coding sequence variations in our cohorts of primary congenital glaucoma, Axenfeld-Rieger syndrome, primary open angle glaucoma, and control subjects (Table 2). Rare *SH3PXD2B* variants were detected in each cohort that were absent from the normal control cohort. One (4.8%) of 21 primary congenital glaucoma subjects carried a Glu396Lys mutation that is located in the third SH3 domain and has a relatively benign blosum62 score of “1.” Two (6.7%) of the 30 Axenfeld-Rieger syndrome patients carried *SH3PXD2B* variations, one patient with Ala431Thr and another with Pro571Leu. Neither of these variants alter known functional domains of SH3PXD2B, however, one variant, Pro571Leu, has a negative blosum62 score of “-3” implying that it may have some effect on the encoded SH3PXD2B protein. *SH3PXD2B* variants were detected in six (4.7%) of 127 POAG patients, including 4 variants (Gly245Arg, Arg356Gln, Gly481Arg, and Ile832Val) that were absent from normal control subjects. Two of these variants (Gly245Arg and Gly481Arg) have blosum62 scores of −2 and Gly245Arg is also located within the second SH3 domain of SH3PXD2B. Finally, five (5.6%) of 89 normal control subjects were found to carry *SH3PXD2B* mutations with blosum62 scores that range from −3 to +1 and none were located in known functional domains. Of note, two of these variants were unique to the cohort of normal control subjects. These data demonstrate that *SH3PXD2B* variants are not a common cause of primary congenital glaucoma, Axenfeld-Rieger syndrome, or POAG. However, it is certainly possible that our research failed to identify disease-causing mutations in *SH3PXD2B* that would be detectable with the power of a study with larger cohorts of patients and controls.

Among the variants identified, Gly245Arg stands out as a possible rare disease-causing variant. In addition to a pathogenic prediction based on blosum62 score [26,43], the change is also predicted to be deleterious by multiple additional algorithms (data not shown), including Sorting Tolerant From Intolerant (SIFT) [44], Polymorphism Phenotyping (PolyPhen) [45], and Align Grantham Variation Grantham Deviation (A-GVGD) [46]. This is significant as it has been previously suggested that there is improved predictive value when all four of these methods are in agreement [47]. There is also biologic evidence suggesting pathogenicity. SH3 domains typically consist of 5 or 6 beta-strands arranged as two anti-parallel beta sheets that essentially form a barrel-like structure mediating protein–protein interactions [48]. The Gly245Arg substitution affects a highly conserved Gly residue within a linker region between beta-strands contributing to a type II beta-turn. Based on an analysis of 266 nonredundant sequences encoding SH3 domains, this Gly is the fifth most highly conserved residue of the 60 constituting a SH3 domain [48]. The residue conservation at this position is thought to be explained by a requirement for the backbone to adopt a left-handed helical conformation for which Gly is strongly favored, both in SH3 domains [48] and in type II beta-turns in general [49]. Though speculative, it is plausible that the Gly245Arg substitution could disrupt folding and ability of the second SH3 domain to participate in protein–protein interactions, thus resulting in a hypomorphic or dominant negative mutation. However, given the rarity of Gly245Arg variant, additional functional experiments would be required to test this hypothesis directly.

One other *SH3PXD2B* variation (Pro826Leu) was also associated with a blosum62 score of −3 that suggests pathogenicity. However, the proline amino acid in SH3PXD2B protein that is altered by this mutation is not strongly conserved across species, nor does A-GVGD suggest that this variation is likely deleterious. Lastly, the Pro826Leu variant has been detected in the exome sequencing project at a frequency of approximately 1% which suggests that it is too common to be a glaucoma-causing mutation. Despite the suggestive blosum62 score, the sum of the available data does not support a disease-causing role for the Pro826Leu variation.

In summary, we previously showed that mutation of *Sh3pxd2b* generates a severe, congenital form of glaucoma in mice [21,22], which suggests that the human ortholog (*SH3PXD2B*) and interacting proteins are also good candidates for causing disease in humans. We tested cohorts of patients with primary congenital glaucoma, Axenfeld-Rieger syndrome, and POAG for *SH3PXD2B* defects and found several rare variants. While analyses of these data were unable to establish a statistically significant link between *SH3PXD2B* and these eye conditions, we have demonstrated that SH3PXD2B is localized to multiple tissues relevant to glaucoma and identified changes warranting future functional studies.
